# Social Patterning in Adiposity in Adolescence: Prospective Observations from the Chinese Birth Cohort ‘‘Children of 1997’’

**DOI:** 10.1371/journal.pone.0146198

**Published:** 2016-01-06

**Authors:** L. L. Hui, Gabriel M. Leung, C. Mary Schooling

**Affiliations:** 1 School of Public Health, Li Ka Shing Faculty of Medicine, The University of Hong Kong, Hong Kong SAR, China; 2 CUNY School of Public Health and Hunter College, New York City, New York, United States of America; Loyola University Chicago, UNITED STATES

## Abstract

**Introduction:**

Low early life socio-economic position is more strongly associated with adiposity among women than men. We examined whether the sex difference of social patterning in general and central adiposity exists before adulthood.

**Methods:**

In Hong Kong’s “Children of 1997” birth cohort, we used multivariable regression to examine the association of parental education, a marker of early life socio-economic position, with body mass index (BMI) (n = 7252, 88% follow-up) and waist-height ratio (n = 5636, 68% follow-up), at 14 years.

**Results:**

Parental education of Grade 9 or below, compared to Grade 12 or above, was associated with higher waist-height ratio z-score particularly in girls (0.30, 95% confidence interval (CI) 0.19, 0.41) compared to boys (0.12, 95% CI 0.02, 0.22) (p for sex interaction = 0.02). Lower parental education was associated with greater BMI z-score in adolescents of locally born mothers, but not adolescents of migrant mothers, with no difference by sex.

**Conclusions:**

Different social patterning in different markers of adiposity may imply different sociological and biological mediating pathways. A stronger association between low early life socio-economic position and waist-height ratio in adolescent girls may indicate sex-specific influences of SEP related early life exposures on central adiposity.

## Introduction

The relation of socio-economic position (SEP) with obesity is complex. Patterns may vary by level of economic development, sex and age.[1–5] In developed settings, obesity often emerges in early life, particularly among those with lower SEP.[6–8] Low early life SEP independently contributes to general obesity[9] and central adiposity[10] in adulthood, sometimes with stronger associations among women than men.[11] In developing countries the social gradient in obesity reverses with economic development. At the early stages of economic development, higher early life SEP is positively associated with obesity but with economic transition a negative association emerges,[3] first among women and later among men,[12] resulting in a combination of a positive relation in men and an inverse relation in women in some settings.[13,14] Social patterns of obesity are less obvious among children in both developed [5] and developing countries [1] where any social gradient in obesity emerges in adolescence.[15,16]

Reasons for the stronger association of low SEP with obesity among women than men, have not been fully elucidated, nor the life stage when such sex difference emerges. Differences in socio-economic patterning of obesity by sex are usually ascribed to more health consciousness, healthier lifestyles and more social pressure to be thin for women than men.[17] On the other hand, biological pathways whereby exposure to infections and limited resources at puberty generate a greater tendency to specifically central obesity among women than men have also been suggested.[14,18] Social patterning of general and central obesity may help distinguish between these hypotheses. However, the evidence in adults is mixed, with some studies finding a stronger association of low SEP with specifically central obesity among women than men,[11,19] but others not.[13,20] To our knowledge little has been done to examine the association of SEP with general and central obesity among adolescents and assessed differences by sex. One study in UK reported no differences by sex in the relation of SEP with waist circumferences at age 12 years.[21]

To clarify the social patterning of obesity and any differences by sex we studied adolescents in Hong Kong. Hong Kong is a recently developed setting, where childhood BMI is not clearly socially patterned,[22] but an association of low adult SEP with higher general and central obesity was observed among women but not men in a nearby older population in Southern China.[14] Here we took advantage of a population-representative birth cohort, “Children of 1997” to examine the association of low parental education attainment, as a proxy of low SEP, with general and central adiposity in adolescents and any differences by sex. Given the literature suggested the association between SEP and adiposity in different setting may vary by different stage of economic development, we also tested whether the association differed by mother’s place of birth, i.e. more developed Hong Kong or rest of China, as a marker of family background.

## Methods

### The “Children of 1997” birth cohort

The Hong Kong “Children of 1997” [23] is a population representative Chinese birth cohort, consisting of 8327 children (99.8% ethnic Chinese) that covered 88% of all births from April 1, 1997 to May 31, 1997. The study was initially established to investigate the impact of second hand smoke exposure on infant health. Families were recruited at their first postnatal visit to the 49 Maternal and Child Health Centres (MCHCs) in Hong Kong, which parents of all newborns are encouraged to attend for free postnatal care, developmental checks and vaccinations until the age of five years. Baseline characteristics were obtained at recruitment using a self-administered questionnaire in Chinese which included parental age and education, birth characteristics (birth weight, birth order, sex and gestational age), infant feeding and second hand smoke exposure. Passive follow-up via record linkage was instituted in 2005 to obtain: (i) weight and height from birth to 5 years from the MCHCs; (ii) annual measurements of weight and height (grade 1 onwards) and bi-annual assessments of pubertal status (grade 1 onwards), physiological well-being (grade 2 onwards) and blood pressure (grade 5 onwards) from the Student Health Service, Department of Health, which provides free annual check-ups for all school students; (iii) hospital discharge records from the Hospital Authority which manages all public hospitals and (iv) death records from the Death Registry. Active follow-up via direct contact was instituted in 2007, with three postal/telephone surveys conducted during 2008 to 2012. Survey III conducted in 2011/12 included self-reported height and weight, and self-measured waist and hip circumference using an enclosed non-extensible measuring tape. About 70% of the cohort participants responded and reported these measurements. Detailed instructions were given to measure waist circumference at the narrowest part of the waist or at the umbilicus, if waist was not obvious, in light clothing paying special attention to ensuring the measuring tape was parallel to the floor. Measurements were taken to the nearest 0.1 centimetres (cm). Implausible values were checked by telephone and 53 implausible measurements, that could not be verified, were discarded. Agreement between self-reported and independently-measured waist circumference among 172 randomly selected participants was satisfactory with a difference of -0.24 cm (95% confidence interval (CI) -0.84cm to 0.37cm) from the independently-measured value. With each survey, missing baseline data and follow-up data were updated and discrepancies between surveys reconciled. This study was reviewed by and received approval from the University of Hong Kong-Hospital Authority Hong Kong West Cluster Joint Institutional Review Board. Informed written consent was obtained from the parents/guardians whose child agreed to participate in the study.

### Exposure–Highest parental education

The main exposure was highest parental education (or “parental education” thereafter) at birth, as a proxy early life SEP. Education is traditionally very highly value in Chinese culture for itself and as a gateway to advancement. Correspondingly, higher education was associated with higher income in the general population in Hong Kong [24] as well as in our birth cohort. Parental education in Hong Kong likely reflects not only material resources but also social, cultural and symbolic capital of the family.[25] Parental education was self-reported at recruitment. As previously, highest parental education was categorized as Grade 9 or below, Grade 10–11 and Grade 12 or above.[22]

### Outcome–adiposity in adolescence

General adiposity at 14 years was proxied by age and sex-specific BMI z-score, calculated from clinically obtained height (nearest to 0.1 cm) and weight (nearest to 0.1 kilogram or (kg)) at the Student Health Service, or if not available by self-report obtained from Survey III (18% at 14 years). We used age (in days) and sex specific z-scores for BMI relative to the 2007 WHO growth reference. We used the akima package in R (version 2.3.1) to interpolate the WHO standards onto a daily scale, so that all z-scores were calculated at exact daily ages. Measurements were not all taken exactly at 14 years; we used the latest available height and weight measurement from age 12.0 years to 14.9 years.

Central adiposity at 14 years was proxied by waist-to-height ratio (WHtR) z-score because WHtR is a better screening tool for cardiovascular disease risk.[26] WHtR was calculated by dividing waist circumference (cm) by height (cm). WHtR were transformed into sex specific z-scores for comparability and ease of interpretation.

Since weight and height for calculating BMI were mainly obtained from the Student Health Service whilst waist circumference and height for calculating WHtR were obtained from Survey III, the age when BMI and WHtR were obtained and the height used for calculating these two indices for the same cohort participant were slightly different.

### Statistical analysis

We used multivariable linear regression to assess the adjusted associations. We tested whether the associations varied by sex from the heterogeneity across strata and the significance of interaction terms. We also tested whether the associations varied by mother’s place of birth (Hong Kong or migrant from mainland China (>80%) and other places), because we previously observed such differences in childhood.[22] Potential confounders considered were sex and for WHtR age at measurement. We did not adjust for other factors associated with parental education and adiposity, such as birth characteristics, because these may represent some of the intervening processes by which SEP is embodied, i.e., these are potential mediators. Given that children of 9–14 years old are at varying stages of puberty, we adjusted for pubertal status in a sensitivity analysis. Timing of puberty was proxied by age- and sex- specific z-score, referenced to local charts,[27,28] for Tanner stages for breast/genital development recorded closest to 12 years of age.

We used multiple imputation to predict missing confounders and exposures, based on a flexible additive regression model with predictive mean matching incorporating data on infant growth, birth weight, gestational age, sex, parity, mother’s smoking during pregnancy, the presence of gestational diabetes and preeclampsia, method of delivery, type of birth hospital, parent’s age at delivery, parents’ height and weight, parents’ occupation, household income, infant feeding, timing of puberty, parents’ place of birth, parental education, the interaction terms of interest (including sex by parental education, mother’s place of birth by parental education, mother’s place of birth by sex and mother’s place of birth by parental education) and the outcomes of interests (i.e., BMI and WHtR.) Parental education and mother’s place of birth was imputed for 3% and 8% respectively. We summarized the results from ten imputed datasets into single estimates with CIs adjusted for missing data uncertainty.[29]

## Results

### Participant characteristics

Of the original 8327 cohort members, as of 30^th^ April 2012, 8285 were alive and had not withdrawn (n = 26 withdrawn), from which 7252 (88%) had information on BMI and 5636 (68%) had WHtR at 14 years. Cohort members excluded were more likely to have parents with more education. BMI was obtained at the mean age of 13.8 years (age ranged from 12.0 to 14.9 years). Mean BMI were 20.0kgm^-2^ for boys and 19.8kgm^-2^ for girls. Self-measured waist circumference was obtained at the mean age of 14.4 years (age ranged from 13.9 to 15.0 years). Mean WHtR was 0.44 for boys and 0.43 for girls. BMI was positively associated with WHtR.

Table 1 shows the participant’s characteristics by highest parental education (Grade 9 or below, Grade 10–11 and Grade 12 or above). As expected parental education were positively associated with family income. Children whose parents had less education were more likely to have mothers born in the rest of China.

**Table 1:** Baseline characteristics by parental education for the Hong Kong ‘Children of 1997’ birth cohort, using imputed data.

	Highest parental education		
Characteristics	≥Grade 12(n = 1870)	Grade 10–11(n = 3144)	≤Grade 9(n = 2238)
Boys (%)	52.3	52.7	51.2
Household income at birth (%)			
1^st^ Quintile	3.6	15.6	41.2
2^nd^ Quintile	4.7	21.5	32.9
3^rd^ Quintile	13.7	24.6	18.6
4^th^ Quintile	25.2	25.6	6.2
5^th^ Quintile	52.8	12.7	1.1
Mother’s place of birth (%)			
Hong Kong	79.0	65.8	35.5
Rest of China/other	21.0	34.2	64.5

### Parental education and adiposity

The association of parental education with BMI z-score at 14 years differed by mother’s place of birth (p for interaction 0.002, Table 2). Parental education had a negative association with BMI z-score among children of Hong Kong born mothers. However the association was in the other direction, although the CIs included no association, among children of migrant mothers. There was no three-way interaction by sex and mother’s place of birth but the positive association of parental education with BMI z-score in children of migrant mothers was stronger in boys (p for sex interaction 0.02).

**Table 2:** Associations of parental education with BMI z-score and WHtR z-score at 14 years in Hong Kong’s “Children of 1997” birth cohort.

	Highest parental education				P for interaction		
	≥Grade 12	Grade 10–11	≤Grade 9	P for trend	mother’s place of birth		
	(n = 1870)	(n = 3144)	n = 2238			sex	both
**BMI z-score**							
**All**	Ref	0.04 (-0.04, 0.11)	0.04 (-0.04, 0.12)	0.29	0.002	0.24	0.67
**Children of Hong Kong born mothers**	Ref	0.07 (-0.01, 0.15)	0.15 (0.04, 0.26)	0.01	-	0.24	-
**Children of migrant mothers from China**	Ref	-0.09 (-0.23, 0.05)	-0.13 (-0.26, 0.01)	0.09	-	0.02	-
**WHtR z-score**							
**All**	Ref	0.08 (0.01, 0.14)	0.21 (0.13, 0.28)	<0.001	0.40	0.02	0.99
**Boys**	Ref	0.04 (-0.05, 0.13)	0.12 (0.02, 0.22)	0.02	0.52	-	-
**Girls**	Ref	0.11(0.02, 0.20)	0.30 (0.19, 0.41)	<0.001	0.61	-	-

Adjusted for sex, age0020(years) at measurements (for WHtR z-score only) and mother’s place of birth (except for stratified models by mother’s place of birth).

The association of parental education with WHtR z-score varied by sex (p for sex interaction 0.02, Table 2). A negative association of parental education with WHtR was stronger in girls than boys. The associations of parental education with WHtR z-score did not vary by mother’s place of birth, and there was no three-way interaction by sex and mother’s place of birth. Similar results were obtained when the timing of puberty was further adjusted in boys and girls together or separately.(data not shown)

For completeness Table 3 shows the joint association of parental education and mother’s place of birth with BMI and WHtR z-score in boys and girls separately. Although the association of parental education with BMI z-score did not differ by sex or by mother’s place of birth and sex, the negative association of parental education with BMI z-score was more obvious among girls of Hong Kong born mothers whilst a positive association of parental education with BMI z-score was more obvious among boys of migrant mothers. On the other hand, girls of lower educated parents had greater WHtR z-score, regardless of mother’s place of birth, while no obvious trends in WHtR by parental education was observed in boys.

**Table 3:** Joint associations of parental education and mother’s place of birth with BMI z-score and WHtR z-score at 14 years by sex in Hong Kong’s “Children of 1997” birth cohort.

	Boys			Girls		
	Highest parental education			Highest parental education		
	≥Grade 12	Grade 10–11	≤Grade 9	≥Grade 12	Grade 10–11	≤Grade 9
**BMI z-score**						
Mother’s place of birth						
Hong Kong	Ref	0.10 (-0.03, 0.22)	0.10 (-0.06, 0.27)	Ref	0.04 (-0.07, 0.14)	0.21 (0.07, 0.34)
The rest of China	0.39 (0.19, 0.60)	0.16 (0.01, 0.31)	0.16 (0.02, 0.29)	0.07 (-0.11, 0.25)	0.15 (0.03, 0.28)	0.08 (-0.04, 0.19)
**WHtR z-score**						
Mother’s place of birth						
Hong Kong	Ref	0.08 (-0.03, 0.18)	0.15 (0.01, 0.28)	Ref	0.09 (-0.02, 0.20)	0.35 (0.21, 0.50)
The rest of China	0.13 (-0.05, 0.30)	0.06 (-0.06, 0.18)	0.16 (0.05, 0.27)	-0.01 (-0.19, 0.18)	0.14 (0.02, 0.27)	0.27 (0.15, 0.38)

## Discussion

In the large and population-representative birth cohort “Children of 1997”, at ~14 years the association of SEP with central adiposity, but not general adiposity, varied by sex, such that lower parental education, a proxy of lower SEP, was associated with higher WHtR specifically among girls. In addition, the association of parental education with BMI differed by mother’s place of birth, such that lower parental education was only associated with greater BMI among children of Hong Kong born mothers but not children of migrant mothers. The differences in the social gradient of markers of central and general adiposity by sex and mother’s place of birth suggest that early life SEP may affect different markers of adiposity through different pathways.

Despite the large sample size and high follow-up rate, there are some caveats in the study. First, attending the Student Health Service for regular health checks and provision of self-measured waist circumferences are voluntary; those excluded due to missing data on adiposity were more likely to have parents with higher education. However 88% of those eligible were included for BMI and 68% were included for WHtR. We have no reason to think that the associations of parental education with adiposity were different among those excluded. Second, we recruited at the well baby clinics soon after delivery. Inevitably the birth cohort did not include a small number of children who stayed in hospital in those early weeks. Third, waist circumference was self-measured and self-reported and may be subject to measurement error. However the validation study found very little bias between self-measured and assessor-measured values. Finally, comprehensive information on lifestyle is not available for the cohort, so potential mediators, such as diet, could not be investigated.

Similar to our observations in the “Children of 1997” cohort in childhood,[22] the relation of parental education with BMI at 14 years differed by mother’s place of birth, representing different living condition of the mothers when they grew up, with a negative association for children of Hong Kong born mothers but not for children of migrant mothers. Whether these associations also differ by sex requires examination in a larger sample. The different social patterning of BMI observed here is similar to difference in the social gradient in BMI reported in a study of adults from 35 European and North American countries, where a negative gradient in BMI was mainly observed, with exceptions in boys in a few lower income countries.[30] Similarly a study in U.S. also observed a negative social gradient in BMI among white adolescents, but not in other ethnic groups.[31] Similar differences in social patterning of adolescent BMI have also been observed among adults in developed and developing countries, where SEP is negatively associated with BMI in developed settings[5] while the relation in developing settings varies by stage of economic development, with the association changing from positive to negative as average income increases.[3] Migrants may be healthier, however this does not explain the positive association of parental education or SEP with BMI in adolescents with migrant mothers. In our birth cohort, mothers born in Hong Kong, compared with migrant mothers who grew up in more limited conditions in the rest of China in 1960s-70s, may have different attitudes to adiposity, particular among those with higher SEP. Such that the same level of SEP may not translate to the same social benefits [31] and the same lifestyle,[16] resulting in the different socioeconomic gradient in BMI by mother’s place of birth. Nevertheless, we cannot rule out the possibility that these differences are due to inter-generational biological processes, such as epigenetic mechanism [32] including DNA methylation which varied by early life SEP.[33]

Lower parental education was associated with greater WHtR z-score particularly among adolescent girls. Although girls had earlier onset of puberty, the sex difference remained after further adjusting for pubertal status in all or in girls only and pubertal timing is not a common cause of parental education and adiposity, suggesting the sex difference in the association was not due to the sex or SEP difference in pubertal development. Such sex difference in the social gradient of central adiposity has been similarly observed in this setting with more marked disparities in central adiposity among women than men,[14] as well as in the West with a stronger negative social gradient in BMI among adolescent girls.[15,30] The negative social gradient of central adiposity in girls is also consistent with evidence from long-term developed settings [21,34] and settings undergoing rapid development.[35] Diet, physical activity, second hand smoking,[36] behavioural [37] or psychosocial risk factors [38] are all possible modifiable causal mediating factors through which parental education specifically affects adolescent adiposity.[37] All of these influences could be stronger in girls, however lifestyle would not explain the inconsistent relation of SEP with general and central adiposity. Alternatively body shape characterizing central adiposity in adolescents is also influenced by sex hormones, with a more gynoid shape in women after exposure to pubertal sex hormones. Lower family SEP maybe potentially linked with intergenerational exposure, such as maternal living conditions, [39] or early life exposures, such as more infections or poorer nutrition, which could affect the gonadotropic axis [18] resulting in lower pubertal sex steroids, leading to more centrally adiposity in girls but not boys.[40,41] However we do not have the measures of central adiposity before puberty to check whether the sex difference in the social gradient of central adiposity only emerged at puberty. In particular whether the tempo or magnitude of pubertal growth affects such social disparity assessed from better markers of fat patterning might provide clarification.

## Conclusions

In the large and population-representative birth cohort “Children of 1997” from a non-Western setting, a stronger inverse association of SEP with central adiposity in adolescence was observed in girls whilst the socioeconomic gradient in general adiposity varied by mother’s place of birth. Early life SEP may exert different influences on different markers of adiposity through different sociological and biological pathways potentially requiring correspondingly different intervention strategies.

## Abbreviations

BMI: Body Mass Index
CI: Confidence Interval
SEP: Socio-economic position
WHtR: Waist-to-height ratio
